# On the Left Ventricular Remodeling of Patients with Stenotic Aortic Valve: A Statistical Shape Analysis

**DOI:** 10.3390/bioengineering8050066

**Published:** 2021-05-13

**Authors:** Salvatore Cutugno, Tommaso Ingrassia, Vincenzo Nigrelli, Salvatore Pasta

**Affiliations:** Dipartimento di Ingegneria (DING), Università degli Studi di Palermo, Viale delle Scienze Ed.8, 90128 Palermo, Italy; salvatore.cutugno@unipa.it (S.C.); vincenzo.nigrelli@unipa.it (V.N.); salvatore.pasta@unipa.it (S.P.)

**Keywords:** left ventricle, aortic valve stenosis, statistical shape analysis

## Abstract

The left ventricle (LV) constantly changes its shape and function as a response to pathological conditions, and this process is known as remodeling. In the presence of aortic stenosis (AS), the degenerative process is not limited to the aortic valve but also involves the remodeling of LV. Statistical shape analysis (SSA) offers a powerful tool for the visualization and quantification of the geometrical and functional patterns of any anatomic changes. In this paper, a SSA method was developed to determine shape descriptors of the LV under different degrees of AS and thus to shed light on the mechanistic link between shape and function. A total of n=86 patients underwent computed tomography (CT) for the evaluation of valvulopathy were segmented to obtain the LV surface and then were automatically aligned to a reference template by rigid registrations and transformations. Shape modes of the anatomical LV variation induced by the degree of AS were assessed by principal component analysis (PCA). The first shape mode represented nearly 50% of the total variance of LV shape in our patient population and was mainly associated to a spherical LV geometry. At Pearson’s analysis, the first shape mode was positively correlated to both the end-diastolic volume (p<0.01, R=0.814) and end-systolic volume (p<0.01, and R=0.922), suggesting LV impairment in patients with severe AS. A predictive model built with PCA-related shape modes achieved better performance in stratifying the occurrence of adverse events with respect to a baseline model using clinical demographic data as risk predictors. This study demonstrated the potential of SSA approaches to detect the association of complex 3D shape features with functional LV parameters.

## 1. Introduction

Aortic stenosis (AS) is a common cardiovascular disease in the developed countries. This type of valvulopathy manifest in 5% of the general population at age of 65 years with increasing prevalence in elderly [1]. Specifically, the population prevalence is estimated to be 12.4% with prevalence of 3.4% of severe AS in those individuals with age of 75 years or older [2]. Stenosis represents a complex systemic disease triggering a degenerative disease development, which is not limited only to the aortic valve but also involves the vascular system and the left ventricle (LV). In fact, the increasing of arterial stiffness is common in patients belonging to the age group most affected by AS [3]. The sum of the outflow tract obstruction due to the calcified aortic valve leaflets and the reduced arterial compliance result in an increasing valvulo-arterial impedance, which in turn represents the factor opposing to ventricular ejection by absorbing the mechanical energy developed by the heart [4]. Several studies have also demonstrated that AS is linked to a stiff dilated aorta [5,6], as assessed by aortic stiffness with either clinical imaging tools [7] or computational models [8,9,10]. To supply the increased requirement of energy, the heart changes his shape and function and this process is well known as cardiac remodeling [11]. Traditional anatomical analysis of shape achieved by parameters measured in 2D (e.g., LV length and diameter) do not fully exploit the abundance of information that current imaging techniques such as cardiovascular magnetic resonance (CMR) or computed tomography (CT) may offer. Conversely, the characterization and quantification of cardiac remodeling by means of statistical tools like statistical shape analysis (SSA) intuitively allows to integrate all anatomical shape information as a visual and numeric mean shape and its principal variations in 3D [12,13,14]. This methodology relies on machine learning as principal component analysis (PCA) to perform data dimensionality reduction and compression and extract key features [15]. There is indeed a growing interest in the research community to apply machine learning in clinical practice to improve clinical imaging diagnosis and tailor patient-specific therapies [16]. Maximization component analysis, surface point distributions and regional manifold learning were also used to generate cardiac atlas as an alternative to SSA. Hence, the application of SSA on a database of LV geometries from patients affected by AS has a double target: (1) quantify the progression of AS and predict the occurrence of adverse events and (2) identify the principal shape features classifying the shape variability and compare them to traditional anatomical and functional parameters. This study sought to determine the patterns of cardiac remodeling in a patient population with different degrees of AS and assess the mechanistic link between shape and left heart function. This was first accomplished by SSA of segmented LV geometries, followed by statistical correlation with functional heart parameters and logistic regression for developing predictive risk models. Findings are therefore discussed.

## 2. Materials and Methods

### 2.1. Study Population

A total of n=86 patients underwent computed tomography (CT) from 2014 to 2019 for valvular diseases were included in this study. Specifically, electrocardiogram-gated cardiac computed tomography (ECG-CT) was performed after initial in-hospital diagnosis of AS as detected by transthoracic echocardiography. Imaging of the heart and proximal vessel was performed with z-resolution of 0.625 mm along the LV short-axis and using a 64 detector–row CT scanner (VCT 64; GE Medical Systems, Milwaukee, WI, USA). In this patient study group, *n* = 30 patients referred for aortic size evaluation were included as a control group because of no-sign of AS and/or regurgitation and preserved LV function. Patient exclusion criteria were uncontrolled history of hypertension, connective tissue disorders, medication or history of coronary artery disease or myocardial infarction. The remaining *n* = 56 patients were stratified into two groups based on the severity of AS (i.e., moderate and severe) according to clinical guidelines on the management of aortic valve diseases of the European Society of Cardiology [17]. To stratify patients in non-complicated versus complicated, the primary endpoint was the surgical repair or the transcatether heart valve replacement of the diseased aortic valve. Specifically, n = 28 patients had stenotic-related complications during the study period. The study was approved by our local ethics review committees, and patients gave informed consent to their inclusion in the study. Table 1 summarizes clinical and demographic characteristics of the patient study group.

### 2.2. Segmentation and Anatomical Measurements

For each patient, ECG-gated cardiac CT images were reconstructed, following a structured reverse engineering approach [18,19], to identify the short- and long-axis of ventricular chambers. Then, the endocardial and epicardial surfaces of the LV wall were segmented by contour lines using a Mimics Innovation Suite (v.20, Materialise, Leuven, Belgium) as done previously by our group [20,21,22,23,24,25,26]. Specifically, the LV geometry was reconstructed at both end-systolic (ES) and end-diastolic (ED) phases, which were defined as the images with the maximum and minimum cross-sectional area, respectively. Segmentations were performed from the apex to the left ventricular base, but not including the complex aortic valve annulus. Once the endocardial and epicardial contours were segmented from image slice, the LV geometry was obtained by the loft protrusion of segmented contour lines using the computer-aided-design software Rhinoceros© (McNeel & Associates, Seattle, WA, USA).

For each patients, we calculated the end-systolic volume (ESV), the end-diastolic volume (EDV) and the stroke volume (SV). The cardiac output (CO) and the left ventricular mass index (LVMI) were also calculated using clinical demographic data.

### 2.3. Statistical Shape Analysis Method

The SSA was performed using a custom algorithm developed in the mathematical language program MATLAB (R2018, MathWorks Inc., Natick, MA, USA) as described by Consentino et al. [12]. To capture all the shape features available, the SSA approach consisted of the pre-processing of segmented LV geometries followed by automatic alignment using rigid registrations and transformations (i.e., isotropic scale, translation and rotation). The algorithm finds the optimal scale, rotation matrix and translation vector minimizing the overall distance between two sets of points with respect to the Euclidean norm. Scale variation between the LV shapes was maintained since heart size is a clinical indicator of disease development. Before shape alignment, LV surfaces were evenly sampled at sufficient resolution to capture all the shape features available for the left ventricular chamber. Specifically, the surface sampling was found optimal for 15,000 Cartesian ($x_{i}$, $y_{i}$, $z_{i}$) points as determined by convergence analysis of the resulting PCA shape modes. Random sampling of the LV model was carried out from low (2000 Cartesian points) to high (25.000 Cartesian points) mesh resolution and then the first shape mode was plotted against the mesh resolution. Convergence was defined when the change of the fist shape mode was <5%.

All LV surfaces were initially aligned to a reference shape that was extrapolated from the patient study group with moderate stenosis as this represents the average shape of our patient population. However, the alignment of LV surfaces to the reference shape can lead to an initial template shape that is quite biased with respect to the initial reference shape. Thus, a new set of shape transformations were developed from the initial template shape to each rigidly aligned shape. The rigid alignment was therefore repeated, using the mean LV surfaces as the reference shape. To reduce bias, the previous steps of rigid alignment, followed by shape transformation and, again, rigid alignment, were repeated a number of times upon the average shape did not change. The so-obtained aligned LV models were therefore used as input for the PCA. The latter is currently one of the most widely used dimension reduction procedures. Using orthogonal transformations, PCA performs a projection of data onto a linear space of maximum variation directions, known as “shape mode” or “mode”. Shape modes are specific aspects of the anatomical LV variation induced by the degree of AS, and help to understand the morphological features that cannot be described by image-based measurements [27]. After PCA, the number of retained shape modes is typically below the number of original variables, yet retains a high percentage of the overall variability in the original population. The first shape mode shows the highest variability in the dataset, and each succeeding mode has the highest residual variance likely showing specific anatomical features of LV shape induced by the degree of AS. In this study, the PCA was carried out using the built-in command of MATLAB after concatenating the coordinate of each LV surface and then assembling all patient data into a matrix. The eigenvectors of the covariance matrix represent the principal component modes, and their corresponding eigenvalues are the proportion of the total variance explained by each mode. The contribution of each mode can be visualized deforming the template from low to high standard deviation values (i.e., ±2σ) of each mode’s deformation vector. Shape vectors numerically denote the contribution that each shape mode has on each LV surfaces, thereby supporting the identification of specific shape features.

### 2.4. Statistical Analysis

Two PCA models for each patient group were considered, the first using the LV surfaces reconstructed at ES and the second using the LV surfaces at ED. After PCA, Pearson correlation was used to explore the relationships between shape modes and functional variables of LV performance. A logistic regression model was also developed to determine which modes is most associated with the occurrence of adverse events (non-complicated versus complicated patients). The weight of shape modes (retained upon 90% of total variance) were used as a predictor for the classification of the patient class. ROC curves were plotted to compute the area under the ROC curve as an index of the predictive value of the regression model. This model was then compared to a baseline model, using clinical demographic data as predictor of adverse events (i.e., heart valve surgery and sudden death) induced by the diseased aortic valve. Statistics was performed using the SPSS software (IBM SPSS Statistics v.17, New York, NY, USA) assuming all probability values considered significant at 0.05 threshold.

## 3. Results

**Figure 1:**
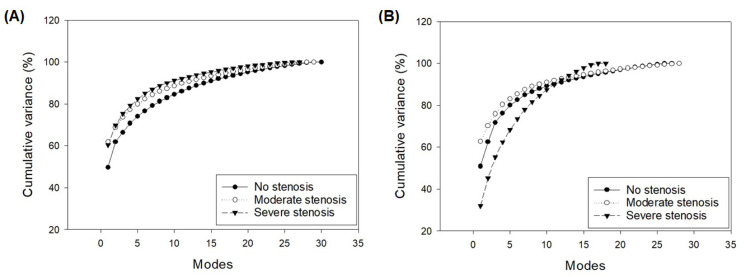
Scree plot of PCA done for all study groups for (**A**) end-diastolic and (**B**) end-systolic cardiac phases.

Figure 1 illustrates the scree plot representing the percentage of total variations of shape modes of LV at both ES and ED. In all cases, the first eight shape modes accounted for nearly 90% of the overall LV shape variability induced by the diseased aortic valve in our patient population.

**Figure 2:**
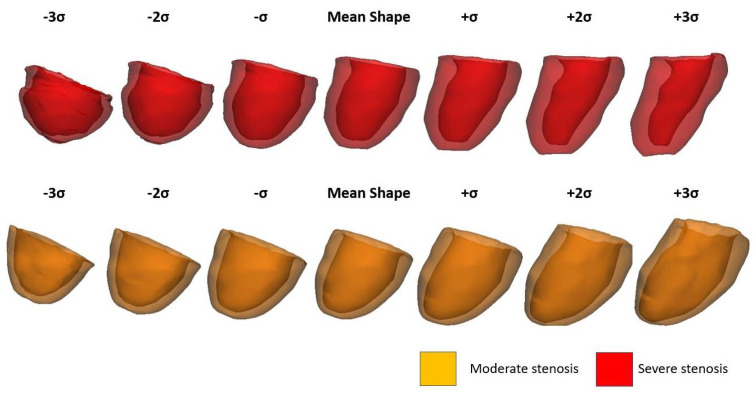
Mode 1 shape variation between −3σ and +3σ for both moderate and severe AS groups at ED configuration.

**Figure 3:**
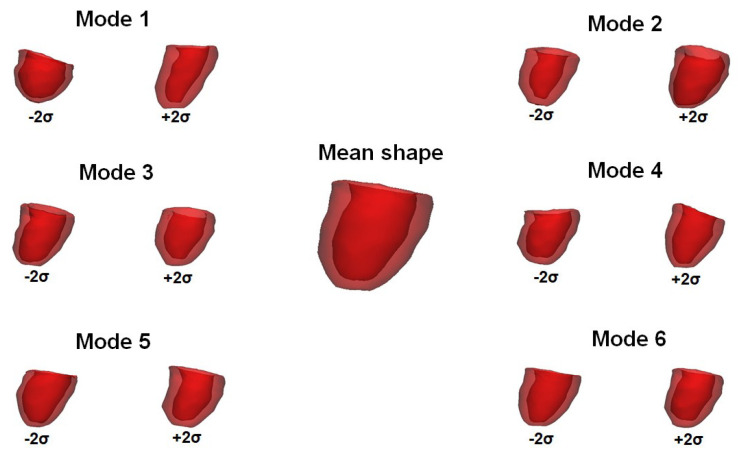
First six shape modes for severe AS study group at ED configuration.

For the patient study groups with moderate and severe AS, Figure 2 describes the first shape mode of PCA variation from significant deformation (±3σ) to less pronounced LV deformation (±σ). The probability of observing significant deformation is <2% while the less pronounced deformation may occur in 25.7% of all cases. It can be observed that the first shape mode is mainly associated to the sphericity of the LV chamber, and this represents nearly 50% of the total variance of LV remodeling. There is no difference in the deformation (±3σ) of the first shape mode for the moderate group versus the severe group. This is expected as the first shape model represents the shape variability of the whole patient population while other modes are generally associated to shape changes that are specific to patient subgroup or disease condition. Indeed, we found that Mode 5 shows difference in the shape between moderate and severe patient groups as characterized by changes in the orientation of the aortic valve plane. Figure 3 illustrates the six shape modes for the patient study group with severe AS. The Mode 2 explains 10% of the overall variance at end-diastole and is primary associated to LV wall thickness. Mode 4 is associated to mitral valve orientation (5%) while Mode 5 to the LV size (1.5%).

**Figure 4:**
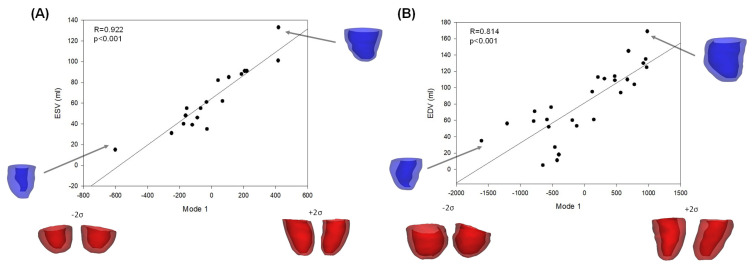
Correlation of Mode 1 with (**A**) end-systolic volume and (**B**) end-diastolic volume for moderate stenosis of LV.

At Pearson’s correlation, we observed a statistically significant positive relationship between the Mode 1 and the ESV (p<0.001, R=0.922, Figure 4A) for the LV with severe stenosis of the aortic valve. The Mode 1 was also positively correlated to EDV (p<0.01, R=0.814, Figure 4B). This suggests that LV remodeling in AS is primary related to change in the LV sphericity. A positive linear relationship was found between the Mode 6 and the cuff-based measurement of the systolic pressure (p=0.012, R=0.820). At ED, the Mode 1was statistically related to the EDV (p<0.001, R=0.851) and LVMI (p<0.01, R=0.599) for the patient group with moderate AS. Differently, the LVMI is negatively correlated to the Mode 3 (p<0.01, R=−0.555), which represents changes in LV length. Significant correlations were also found between shape modes and functional LV performance. For severe stenosis, we found that Mode 6 was positively correlated with the cuff systolic pressure (*p* = 0.012, *R* = 0.820) while Mode 8 was negatively correlated with the diastolic pressure (*p* < 0.001, *R* = −0.917). For moderate stenosis, Mode 5 was positively correlated with the CO measured by echocardiography (*p* = 0.029, *R* = 0.500).

**Figure 5:**
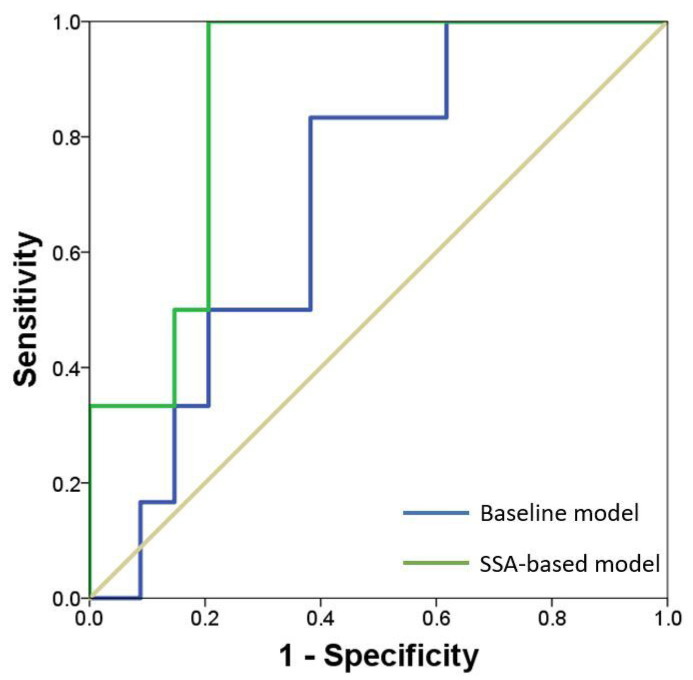
ROC curves for predicting the risk of valvular repair using a model based on principal shape modes as compared to the baseline model based on clinical demographic data.

In order to develop a prognostic model to estimate the probability of repair of diseased aortic valve, a logistic regression based on shape modes retained upon 90% of shape variability was performed. Specifically, the shape modes computed at ES and ED were combined together into one vector. Then, this model was compared to a baseline model considering clinical demographic data as predictors of AS repair. The follow-up period was 3.5 years for control group and 4.6 years for the patient study group with aortic stenosis. The coefficients (β), standard error, *p*-values, standardized coefficients and odds ratios (OR) were computed for each model, see Table 2. The baseline model evinced that the patient age, sex and stroke volume were statistically significant predictors of the disease. The PCA-related model also showed that most shape modes were significantly associated with the disease (except Modes 3, 7 and 8) while the β values demonstrated that Mode 1 has greater effect in the classification model at ED. The Odd ratios were relative indicators of the effect of the shape variability between complicated and non-complicated AS patients, with the Mode 1 and 2 (with OR > 1) showing higher odds of valvular repair than other shape modes. Finally, Receiver Operating Characteristic (ROC) curves highlighted that the principal shape modes of LV geometrical variation can predict with high sensitivity and specificity the probability of adverse events (AUC=0.873), as compared to the baseline model built with demographic data (AUC=0.696, see Figure 5).

## 4. Discussion

Patients with AS have concomitant LV remodeling and proximal arterial disease. Clinicians should be therefore aware that the degenerative process involves not only the aortic valve but also the left heart. Under this condition, the LV undergoes increased load so that early detection and accurate estimation of LV remodeling is crucial to improve risk stratification and clinical decision-making. In this study, SSA was proposed to investigate the LV geometrical changes under different severities of AS to demonstrate the potential of shape analysis for discovering previously unknown anatomic features that may ultimately improve prophylactic intervention. The methodology consisted of three steps: (1) segmentation of LV wall, (2) PCA of the aligned LV models with respect to a template, (3) detailed quantification of the association between functional parameters and LV shape modes using Pearson’s correlation and logistic regression. We speculated that specific shape features characterize the LV remodeling induced by different degrees of AS and may be predictors of adverse events (i.e., repair of diseased aortic valve). Findings supported this hypothesis, with extracted shape modes statistically associated with the disease status. Sphericity represented the main variation in the LV shape and had a strong correlation with both ESV and EDV, thereby suggesting LV impairment with severe AS. Moreover, the PCA-related model built with shape modes performed better than traditional clinical metrics of remodeling based on demographic data and functional parameters (i.e., age, sex and stroke volume). This SSA approach can be therefore adopted as a clinical tool to extract 3D shape biomarkers characterizing the patterns of LV remodeling induced by the stenotic aortic valve. We described how SSA has the potential to discover previously unknown 3D shape biomarkers in a challenging patient population and to develop predictive model integrating diagnostic imaging and mathematical modeling to improve decision making. Using longitudinal imaging data, the SSA can be used to track individual patients over time and identify LV geometries who are likely evolving towards adverse outcomes and thus deserve a more aggressive management. However, further studies are needed to confirm the prognostic potential of novel 3D shape biomarkers of LV wall in the setting of AS.

In failing heart, ventricular size and shape are the most common geometric aspects of the LV wall. Quantitative post-mortem anatomic observations revealed a more spherical shape of dilated hearts after myocardial infarction [28]. This clinical evidence related to the increased sphericity in failed hearts has led to the development of the novel sphericity index as way to identify early patterns of LV remodeling [29]. It is well known that theraupetic strategies have to interrupt the vicious cycle of progressive cardiac dilatation and remodeling in order to reverse the natural history of the failing heart. After myocardial infarction, an increase in heart size is considered a predictor of mortality while LV sphericity is associated with decreased survival rate [30]. Regenerative medicine approaches have also demonstrated that the re-shaping of a failing LV towards a more parabolic shape can reduce intramural wall stress and thus invert the remodeling pathway [31]. We here extended the observations of spherical LV geometries in patients with AS using quantitative shape features and association with functional parameters. This association was not known previously, which is why we considered the extracted 3D shape modes as potential and novel morphological biomarkers of LV remodeling. We also aimed to raise the awareness on the importance of detecting outlying shapes associated to outlying functional behavior as shown by the correlation of Mode 1 with both ESV and EDV (see Figure 4). One reason for this result may be that our patient study group with severe AS had preserved LV function in spite of a high transvalvular pressure gradients induced by the stiff AS valve. From a clinical perspective, this highly insidious situation represents a challenging clinical entity because the aortic stenosis may appear less severe if graded using only the transvalvular pressure gradient. Early diagnosis and accurate estimation of the severity of AS is therefore of primary importance, although this is challenging to be assessed with conventional echocardiographic modalities and morphometric measurements.

For this reason, SSAs have demonstrated the potential to reveal new measures of geometry and function as they integrate all anatomical shape information intuitively as a visual and numerical mean shape and its variation in 3D [16]. Such atlas-based models have been adopted to determine shape-related differences of the diseased left heart. Lewandowski et al. [32] evinced that individuals born prematurely had a unique LV geometry and significant reductions in systolic and diastolic functional parameters as compared to matched controls. In myocardial infarction, Zhang and collaborators [33] performed a SSA for characterizing the LV remodeling and demonstrated the benefit of shape descriptors for predicting the risk of adverse events. Ardekani et al. [34] determined the statistical variations of LV surface deformation in hypertensive and hypertrophic heart disease. Procrustes analysis was also adopted to extract shape features of the motion in normal LVs [35]. Although SSA methods were widely adopted by many research group, clinical applications have been recently explored for the aortic arch shape [36]. This study therefore represents a further step towards the use of SSAs to provide unique shape features for the clinical evaluation of AS over the demographic variables.

The study has the disadvantages of a retrospective design, with limited and an uneven number of patients in each subgroup. Indeed, the complicated study group included patient with moderate valve stenosis who underwent surgery because of concomitant cardiac or vascular complications. This has likely affected the statistical analysis but aortic stenosis is often associated to detrimental effects on LV function and a loss of arterial elasticity. Thus, aortic stenosis cannot be considered as an isolated disease. There was also not any significance on the left ventricular hypertrophy (i.e., increase in LV mass) in the patient group with severe aortic stenosis as compared to that of patient with moderate stenosis and controls. In the logistic regression model, gender differences were included because of the general consensus on the high risk of cardiovascular events in the male versus female population, and this has likely influenced model output. Most importantly, the segmentation of LV geometry did not include the stenotic aortic valve as this was not seen at CT scan. This is in part justified by the fact that the adaptation of the LV wall to the disease progression is mainly accompanied by changes in ventricular size and shape. Findings presented in this study are meanly meant to demonstrate the potential of the proposed SSA approach by studying the association of complex 3D shape features with functional echocardiographic parameters. In patients with AS, our approach applied to a larger and heterogonous patient cohort could help to understand the efficacy and predictive capability of LV shape modes for early diagnosis of the valvulopathy.

## 5. Conclusions

In this study, the complex shape features of the left heart chamber were extracted by SSA and then correlated to clinical and functional data of patients with different degrees of AS. This was performed to shed light on shape and function as well as improve patient risk prediction using a detailed 3D shape analysis at minimal human intervention.

## Figures and Tables

**Table 1:** Demographic and clinical data of study population divided by groups.

	No Stenosis	Moderate Stenosis	Severe Stenosis	*p*-Value
Age (years)	60.14±10.11	60.93±12.03	69.11±12.82	0.007 *
BSA ($m^{2}$)	2.04±0.22	2.01±7.74	1.90±0.15	0.679
BMI ($\mathrm{kg}/m^{2}$)	28.76±4.19	24.55±9.64	27.64±3.51	0.167
Heart Rate (bpm)	73.19±11.79	68.09±11.97	75.38±11.04	0.237
SP (mmHg)	132.59±10.95	134.25±19.26	139.38±8.51	0.600
DP (mmHg)	76.74±8.53	77.38±12.88	72.88±5.90	0.546
MAP (mmHg)	92.44±6.78	93.33±9.56	91.19±8.58	0.636
SV (ml)	77.92±25.27	80.52±26.45	82.86±28.27	0.870
CO (ml/min)	5624.83±2170.20	5286.29±2283.74	6075.86±2427.20	0.752
TA flow (m/s)	1.38±0.49	1.81±0.59	2.52±1.15	<0.001 *
LVMI ($g/m^{2}$)	101.73±44.97	112.32±60.89	91.80±60.33	0.293
EDV (ml)	76.18±26.10	82.89±33.38	81.44±42.34	0.710
ESV (ml)	58.28±22.13	60.70±28.97	64.33±29.07	0.865
EF (%)	63.61±11.04	64.82±4.90	60.01±17.23	0.409
ΔP (mmHg)	8.5±7.9	14.4±10.5	35.0±26.1	<0.001 *
Sample size	30	29	27	

Note: BSA = body surface area; BMI = body mass index; SP = Systolic Pressure; DP = Diastolic Pressure; MAP = mean arterial pressure; SV = stroke volume; CO = cardiac output; TA flow = transaortic flow; LVMI = left ventricular mass index; EDV = end-diastolic volume; ESV = end-systolic volume; EF = ejection fraction; Δ*P* = transvalvulare pressure gradient; * significant difference (*p* < 0.05).

**Table 2:** Logistic regression analysis of the shape modes retained upon 90% of shape variability.

Parameter	Coefficient	Standard Error	SC	OR	OR 95%CI	
Constant	−9.507	2.278				
Age	0.130	0.330	0.356	0.799	0.260	2.264
Sex	2.180	1.031	0.651	1.039	0.986	1.082
Mode 1 *	0.024	0.002	2.65	1.024	1.019	1.035
Mode 2 *	−0.012	0.003	−0.589	1.011	0.984	0.992
Mode 3	−0.002	0.004	−0.091	1.038	1.035	1.038
Mode 4 *	0.056	0.002	0.228	1.007	1.001	1.013
Mode 5	0.019	0.007	0.257	1.021	1.006	1.034
Mode 6 *	−0.012	0.008	−0.145	0.978	0.961	1.003
Mode 7	−0.019	0.007	−0.247	1.020	1.066	1.034
Mode 8	−0.031	0.005	−0.913	1.010	1.000	1.030

Note: SC = Standardized coefficient; OR = Odd ratio; * indicate *p* < 0.05.

## Data Availability

Data is contained within the article.
